# Annotations

**Published:** 1896-11-14

**Authors:** 


### Annotations.

### Alcohol and Digestion.

The value, or valuelessness, of alcohol as a food, or as an aid to digestion, is an old controversy, and is held by some to be as difficult of settlement as any question which admits of scientific handling can well be. Nevertheless, as the methods of science improve, trustworthy and quite convincing results may be with certainty hoped for. It is well known that pieces of solid beef, cut up fine, can be reduced to a liquefied condition by the action of an artificial gastric juice. It ought not, therefore, to be very difficult, and it is not, indeed, difficult at all, to add alcohol, in varying quantities, to a known digestive fluid, and to test the result on all sorts of substances which may be presented for artificial digestion. Nor is there any mystery about such processes. Any person with a little common sense may employ them for his own satisfaction. In the physiological department of Yale University, Drs. Chittenden and Mendel have recently made a series of such experiments, and have published the results obtained. According to these two American doctors, alcohol in small quantities distinctly aids the solution of solid matters when added to artificial digestive fluids. On the other hand, in large, or even considerable quantities, it definitely checks solution. The experiments of the two physiologists were made with absolute alcohol, with rye whiskey, with brandy, rum, gin, and other substances. These results coincide exactly with a great deal of common experience. There is no doubt whatever that some persons can digest their food more comfortably, more quickly, and more completely when they take a little alcohol than they can without it. There appears to be equally no doubt that certain other persons can digest their food with all needful comfort, celerity, and completeness without alcohol. So far, then, as the mere solution of food in the stomach is concerned, the very obvious conclusion of the matter would appear to be this—that those persons who are benefited by alcohol should certainly take it, whilst those who can do without it should settle the question of taking or not taking it on any special grounds which may be peculiar to themselves.

### The Administration of Anæsthetics.

An article which recently appeared in The Hospital on "Instrumental Aids to the Administration of Anæsthetics" has drawn upon us a considerable amount of correspondence, partly dealing with the relative advantages of chloroform and ether, a subject which for the present we must put on one side, and partly with the much more urgent question of the best mode of administering chloroform. We say this is the more urgent question, because until some general consensus is arrived at as to the best and safest method of administration, it is impossible to judge as to the relative advantages of the two anæsthetics. It is a startling thing to hear, as we do, that forty-eight deaths from chloroform, or occurring during its administration, have already taken place this year, especially when we bear in mind the possibility of this result arising from an overdose. The questions at once occur, What is an overdose? and How is overdosing to be prevented? As to the first, we are afraid that no definite answer can at present be given. It is probable that the limit of safety depends largely upon idiosyncrasy. What is certain, however, is that the actual amount of chloroform which must be absorbed into the blood, to produce its effects, is very small compared with the quantity commonly used in the open method of administration; and one cannot but see, and seeing one cannot but regret, that the vast amount of experience which is being daily gained in the administration of chloroform is being practically thrown away, so far as the acquisition of accurate knowledge is concerned, in consequence of the common lack of all means of knowing what dose is being given in each case. In regard to the prevention of deaths from chloroform, we would not speak so positively as some appear inclined to do as to the safety of small doses well diluted. But at the same time we would say that even the first step towards safety has not been taken so long as administrators do not know how much chloroform their patients are getting. An unbroken record of some thousands of cases in which chloroform had been administered by methods involving measured and minimum dosage would go further than any quantity of laboratory experiments in solving the chloroform problem.

### Is the Lady Guardian a Failure?

According to the report for 1895-6, just issued, of the Local Government Board, lady guardians have been elected in considerable numbers during the last few years, and it is now possible to form some kind of estimate of their utility and success in their new positions. Many people have raised theoretical objections to the lady guardian, and it has been noted as a fact, not without interest, that the objectors have been found chiefly in the ranks of married men. The "proof of the pudding," as we so often insist here, "is in the eating"; and we are now furnished with a mass of facts, gathered from impartial sources, and stamped with the official authority of a Government department. More than this we need not ask for. What then is the testimony of the facts which bear upon the question of the success or failure of ladies as guardians of the poor? Speaking broadly, it may be stated that they have been "victorious all along the line." Most of the Local Government inspectors comment in some way upon the question; and some speak with marked decision. Mr. Davy thinks they have been "most useful, and will become more useful still as more experience is gained." Mr. Preston Thomas holds that they are very valuable, but he is inclined to the view that the "warmth of their sympathies sometimes leads them astray," and especially in the matter of outdoor relief. These, it must be remembered, are official opinions; and it is very seldom that official sympathy itself goes "astray" by excess of "warmth." Perhaps, if the lady guardians, of whom Mr. Davy and Mr. Preston Thomas writes, were invited to express their opinions both of the official inspectors and of their fellow guardians of the sterner sex, it might appear that there was some falling short of absolute perfection even in the capacity and utility of the male creature. Be that as it may, it is very satisfactory to know that the lady guardian is amply justifying her appointment, and that the lot of the women and children whose misfortune it is to have the Poor Law for a parent, is sensibly ameliorated and improved by the new condition of things.

